# Interactions between Tryptophan Metabolism, the Gut Microbiome and the Immune System as Potential Drivers of Non-Alcoholic Fatty Liver Disease (NAFLD) and Metabolic Diseases

**DOI:** 10.3390/metabo12060514

**Published:** 2022-06-02

**Authors:** Charlotte Teunis, Max Nieuwdorp, Nordin Hanssen

**Affiliations:** Department of Internal and Vascular Medicine, Amsterdam University Medical Center, 1105 AZ Amsterdam, The Netherlands; m.nieuwdorp@amsterdamumc.nl (M.N.); n.m.j.hanssen@amsterdamumc.nl (N.H.)

**Keywords:** NAFLD, MAFLD, metabolic disease, gut microbiota, tryptophan metabolism

## Abstract

The prevalence of non-alcoholic fatty liver disease (NAFLD) is increasing and therefore is its burden of disease as NALFD is a risk factor for cirrhosis and is associated with other metabolic conditions such as type II diabetes, obesity, dyslipidaemia and atherosclerosis. Linking these cardiometabolic diseases is a state of low-grade inflammation, with higher cytokines and c-reactive protein levels found in individuals with NAFLD, obesity and type II diabetes. A possible therapeutic target to decrease this state of low-grade inflammation is the metabolism of the essential amino-acid tryptophan. Its three main metabolic pathways (kynurenine pathway, indole pathway and serotonin/melatonin pathway) result in metabolites such as kynurenic acid, xanturenic acid, indole-3-propionic acid and serotonin/melatonin. The kynurenine pathway is regulated by indoleamine 2,3-dioxygenase (IDO), an enzyme that is upregulated by pro-inflammatory molecules such as INF, IL-6 and LPS. Higher activity of IDO is associated with increased inflammation and fibrosis in NAFLD, as well with increased glucose levels, obesity and atherosclerosis. On the other hand, increased concentrations of the indole pathway metabolites, regulated by the gut microbiome, seem to result in more favorable outcomes. This narrative review summarizes the interactions between tryptophan metabolism, the gut microbiome and the immune system as potential drivers of cardiometabolic diseases in NAFLD.

## 1. Introduction

Non-alcoholic fatty liver disease (NAFLD) affects >25% of the population worldwide [1]. As shown by several prediction models the incidence of NAFLD, and therefore the burden of this condition, will increase even further in upcoming years, and this is often attributed to the obesity pandemic [2,3]. NAFLD is defined as hepatic steatosis, confirmed by imaging or histology, and no evidence of a secondary cause (e.g., viral or auto-immune hepatitis, alcohol or medication). Histologically, NAFLD can be divided into non-alcoholic fatty liver (NAFL) and non-alcoholic steatohepatitis (NASH), the latter increasing the risk for progression to cirrhosis [4] and hepatocellular carcinoma [5]. Furthermore, NAFLD and NASH are linked to a range of cardiometabolic conditions, such as type 2 diabetes mellitus (T2DM) and cardiovascular disease. Given its rising prevalence, and its association with a wide range of chronic diseases, a better understanding of the pathogenesis of NALFD and its potential progression towards NASH and cirrhosis is urgently needed.

Although obesity seems to be the major driver of NAFLD [6], the pathogenesis for NAFLD and especially its progression towards NASH is proposed as a ‘multiple hit model’, wherein an interaction between multiple pathways leads to liver injury, inflammation and fibrosis [7,8]. Currently, the main established pathophysiological pathways in NAFLD are considered to be lipotoxicity, leading to apoptosis and therefore instigating an inflammatory response. Over time, continuous lipid loading leads to oxidative stress with fibrogenesis as a final result [7]. The correlation between NAFLD and metabolic diseases such as T2DM, obesity and cardiovascular disease (e.g., hypertension, hypertriglyceridemia and atherosclerosis) have been well established [6,9,10]. In a population of individuals with a positive ultrasound for steatosis, 67.5% was obese, 68.2% had hypertension and 26.3% was diagnosed with diabetes [11]. Conversely, in patients with diabetes, 56% meet the criteria for NAFLD [12].

In addition, emerging risk factors such as the gut–liver axis have been well studied and this is one of the pathways associated with liver inflammation in NAFLD [13]. The gut–liver axis is currently understood as a perturbation of gut microbial composition, and a disruption of the intestinal barrier, increasing gut barrier permeability. Consequently, this leads to the translocation of microbes and microbial products, such as lipopolysaccharide (LPS) and other metabolites into the portal bloodstream, directly targeting the liver [14,15,16]. LPS binds to toll like receptor 4 (TLR4) activating the innate immune system in the liver, especially Kupffer cells and infiltrating monocytes/macrophages [16,17]. Combined with activating the MyD88/NF-kB cascade, the binding of LPS to TLR4 induces the release of pro-inflammatory cytokines such as interleukin 6 (IL-6), IL-17, tumor necrosis factor α (TNF-α) and interferon γ (INF-γ), eventually leading to the release of fibrogenic factors [8,18,19].

The gut microbiome is, in addition to its symbiotic functions by regulating the inflammatory and immunological tone of the host [20] and prevention of pathogenic overgrowth of harmful microbes such as Clostridium difficile, increasingly compared to an endocrine organ [21]. Herein, the gut microbiome produces a range of metabolites that interact with specific receptors, altering the phenotype of the host. In fact, due to new insights in the pathophysiology of NAFLD and its increasing incidence, an international consensus panel recently proposed to change the term NAFLD to metabolite associated liver disease (MAFLD) and adjust the diagnostic criteria to evidence of hepatic steatosis combined with obesity or T2DM or signs of metabolic dysfunction [22]. A key pathway of interest in this context is the production of tryptophan metabolites by either the host or the gut microbiome [23].

Lower tryptophan levels and increased tryptophan related enzymes (e.g., indoleamine 2,3-dioxygenase 1 and 2 (IDO1 and IDO2) and tryptophan-2,3- dioxygenase (TDO)) and downstream metabolites have been linked to increased metabolic inflammation and fibrosis [24,25]. In NAFLD, particularly indole pathway metabolites, converted by the gut microbiome, have been shown to reduce inflammation via the NF-kB pathway and several other metabolites have been shown to reduce the production of cytokines such as IL-22 and modulate the innate immune system [26]. These pathways are of potential importance as perturbation of the tryptophan metabolism may be a relatively easy therapeutic target in the context of NAFLD/NASH, either by dietary strategies, probiotics (containing microbiota that metabolize tryptophan) or microbiota derived tryptophan metabolites with favorable properties (a concept referred to as postbiotics). In this review we provide an overview of the physiology of tryptophan metabolism, considering both host metabolism as well as microbial metabolism. Due to the intrinsic relation of tryptophan metabolites and related enzymes with the gut microbiome and the immune system, we speculate that disturbances in tryptophan pathways contribute to the development NAFLD, as well to the cardiometabolic conditions that are thought to precipitate NALFD. This is of major interest as NALFD and cardiometabolic diseases are exceedingly common conditions. Therefore, a better understanding of tryptophan physiology, perturbations in tryptophan metabolism due to metabolic dysregulation and its potential impact on metabolic diseases is of vital importance.

## 2. The Physiology of Tryptophan Metabolism and Tryptophan Metabolites

### 2.1. Tryptophan Intake, Absorption and Elimination

**Figure 1:**
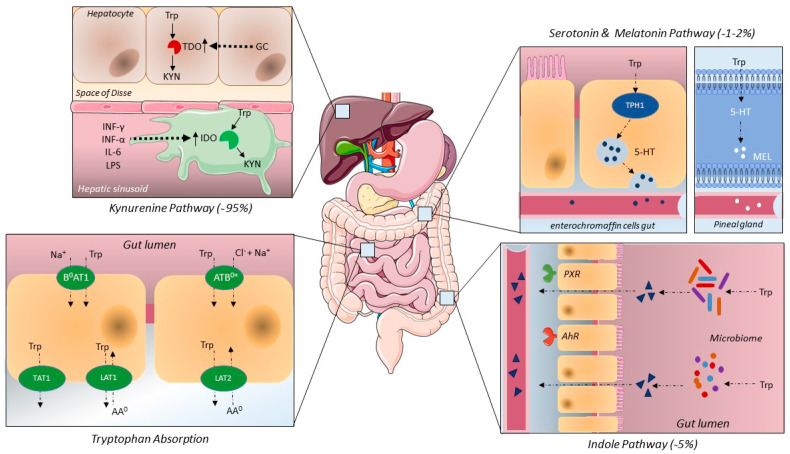
Tryptophan uptake and metabolism. Abbreviations: 5-HT; serotonin, AA^0^; neutral amino acid, AhR; aryl hydrocarbon receptor, GC; glucocorticosteroids, IDO; idoleamine 2,3-dioxygenase, INF; interferon, IL; interleukin, KYN; kynurenine, LPS: Lipopolysaccharide, MEL; melatonin, PXR; Pregnane X receptor, TDO; tryptophan 2,3-dioxygenase, TPH1; tryptophan hydroxylase 1, Trp; tryptophan.

Tryptophan is one of the nine essential amino acids and is found in relative abundance in turkey, chicken, milk, tuna, nuts and bananas. The average intake of tryptophan in adults is about 900–1000 mg/day, where recommended daily intake would be around 3.5–6.0 mg/kg [27]. Tryptophan, together with phenylalanine and tyrosine is called an aromatic amino acid due to a benzene ring sidechain, which in tryptophan specifically consists of indole [28]. Tryptophan catabolism constitutes three main pathways; the kynurenine pathway, which takes up about 90–95% of tryptophan metabolism, the serotonin/melatonin pathway, which is about 1–2% percent of tryptophan metabolism and 5% is used in the indole pathway [27,29]. After ingestion most proteins and amino acids are absorbed in the small intestine (see Figure 1) [29]. Dietary intake of amino acids does not seem to affect amino acid concentrations in the cytosol or blood, but ingestion of carbohydrates or protein does alter 5-hydroxyindoleacetic acid (5HIAA) availability in the brain, suggesting a tissue dependent control of tryptophan concentrations [30].

Being a neutral amino acid, tryptophan is absorbed by ATB0+ (SLC6A14, a symporter using 2Na^+^/1Cl^−^) and B0AT1 (SLC6A19, a symporter using 1Na^+^) at the apical membrane of the small intestine and is excreted in to the portal circulation via the basolateral membrane by TAT1 (SLC16A10, a uniporter) or LAT1-4F2hc (SLC7A5-SLC3A2, an antiporter) and LAT2-4F2hc (SLC7A8-SLC3A2, an antiporter) [31,32]. These amino acid transporters are also referred to as system B+ (B0AT1/B0AT2), System B0+ (ATB0+), system L (LAT1-4F2hc/LAT2-4F2hc) and system T (TAT1) [33]. These transporters often need to associate with other molecules such as angiotensin converting enzyme 2 (ACE2), CD98/CD147 [34] or aminopeptidase N [35]. Association with other proteins is thought to be necessary for transporter insertion in the cell membrane, to increase substrate supply or to modulate transporter activity [34]. Factors influencing the expression of these receptors and proteins are to date not completely elucidated. Once absorbed into the circulation, tryptophan is the only amino acid to bind to albumin (75–90%) [27,36]. Factors that negatively influence the amount of tryptophan bound to albumin are low albumin concentrations, certain drugs and non-esterified fatty acids [37].

The same amino acid transporters that are located in the gut are also present in the kidney. On the luminal brush boarder of the proximal kidney tubule there is an abundant expression of B0AT1 dependent on collectrin, in contrast with ACE2 in the intestine. On the basolateral membrane, TAT1 is often collocated with LAT2-4F2hc and drives neutral amino acids into the extracellular space [38,39]. Tryptophan that is not absorbed in the small intestine will be used as an energy source for microbiota in the colon, the indole pathway, serotonin synthesis and for a small portion of the extrahepatic kynurenine pathway [29]. 

### 2.2. The Kynurenine Pathway 

**Figure 2:**
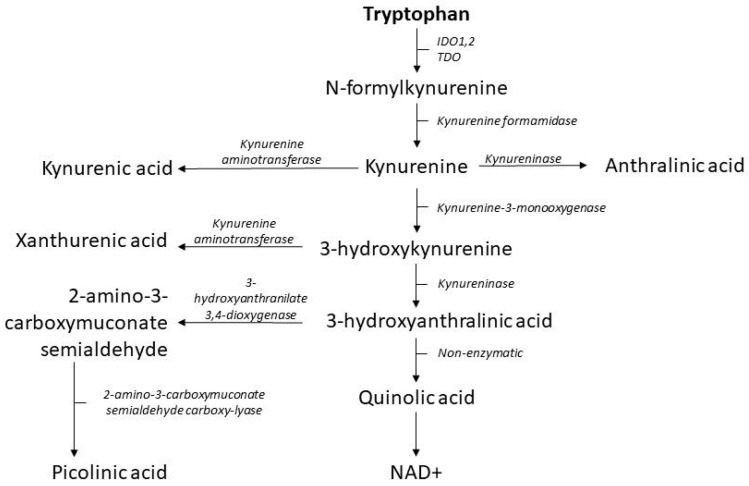
Kynurenine pathway Abbreviations: IDO; indoleamine 2-3-dioxygenase, TDO; tryptophan 2,3-dioxygenase.

The kynurenine pathway is the main catabolic pathway of tryptophan (see Figure 2). Once absorbed into the circulation, about 90% tryptophan will be metabolized by IDO1, IDO2 and TDO into *N*-formylkynurenine [40]. This is the first and rate-limiting step of the kynurenine pathway [30], resulting in the subsequent production of nicotinamide adenine dinucleotide (NAD), kynurenine, kynurenic acid (KA), xanthurenic acid (XA), picolinic acid (PA) and anthranilic acid (AA). 

TDO is mainly expressed in the liver, but also in brain tissue and activity is upregulated by corticosteroids. Intriguingly, some bacterial species express TDO such as *Pseudomonas Aeruginosa* [29]. Compared to IDO, TDO has a higher affinity with tryptophan and exclusively converts tryptophan [41]. One study showed that several metabolites of the indole pathway are inhibitors of TDO, but that these metabolites do not inhibit IDO [42]. IDO has an affinity with multiple substrates and is expressed throughout the body in epithelial and endothelial cells as well as in monocytes, macrophages and vascular smooth muscle cells [43]. IDO1 activity is strongly induced by INF-γ, although other pro-inflammatory molecules are also associated with higher IDO-activity such as INF-α, INF-β, IL-6 and LPS [43,44]. IDO-activity is often measured by the kynurenine/tryptophan-ratio. Yet, this is up for debate since other factors such as TDO activity and the amount of tryptophan bound to albumin is not taken into account when applying this ratio [37]. IDO2 has a low enzymatic activity compared to IDO1 and TDO. The regulation and mechanisms of IDO2 are not completely elucidated but IDO2 seems to have distinct properties from IDO1 [45,46]. 

The regulation of downstream enzyme activity and the effects of downstream metabolites are extensively reviewed in several papers [37,40,47,48,49]. These metabolites are associated with a wide range of diseases including NAFLD, diabetes and cardiovascular disease and will be discussed later in this review.

### 2.3. Indole Pathway 

**Figure 3:**
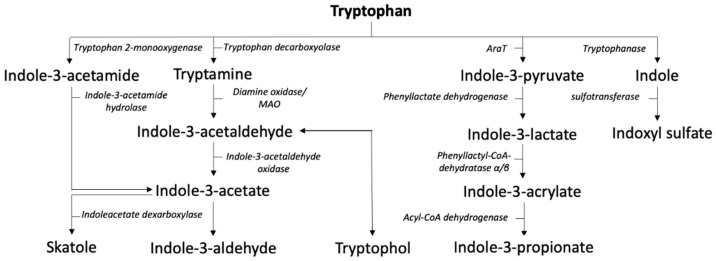
Indole pathway. Abbreviations: AraT; aromatic amino acid aminotransferase, MAO; monoamino oxidase.

Non-absorbed dietary derived tryptophan is transported towards the colon, where the highest concentrations of tryptophan are measured in the distal colon. Here, the indole pathway is regulated by the gut microbiome and results in different indoles such as indole-3-acetate (IA), indole-3-propionate (IPA) and skatole (3-methylindole) (see Figure 3) [50]. The enzymes involved in forming these metabolites are produced by different gut bacteria such as Clostridium spp, Bacteroides spp and Peptostreptococcus spp. A complete overview of species associated with tryptophan degradation was recently given by Roager et al. [51]. The conversion of indole to indoxyl sulfate, however, occurs in the liver and is mediated by cytochrome P450 and sulfotransferase [52,53]. The measurement and concentrations of these metabolites differ significantly depending on the sample in which is it measured (e.g., in brain tissue, cerebrospinal fluid, saliva, plasma and feces) [54]. The kinetics of indole absorption and distribution are not fully understood. 

Bridging microbial metabolites and inflammation are several human receptors affecting the immune system such the aryl hydrocarbon receptor (AhR) and the pregnane X receptor (PXR) [55]. These receptors are linked to inflammation and may therefore indeed mediate some aspects of the gut–liver axis.

**Figure 4:**
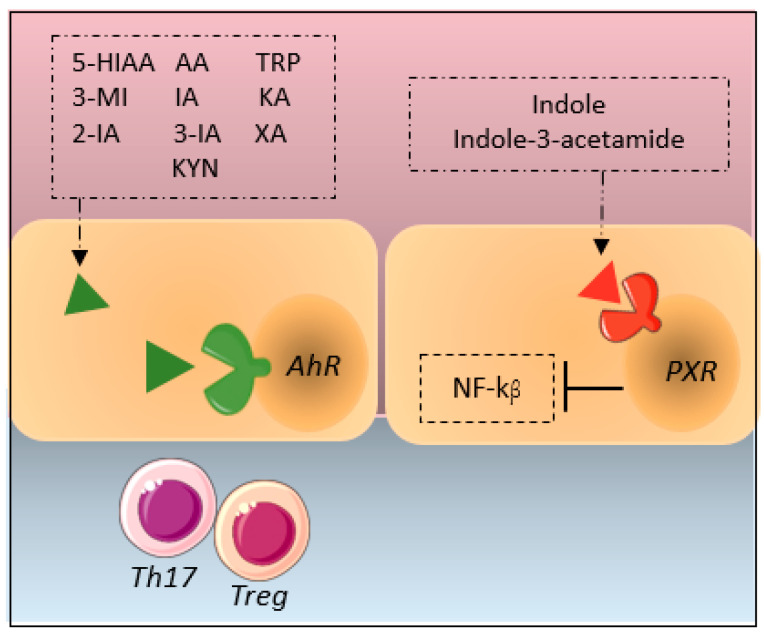
Interactions of tryptophan metabolites with innate immune receptors. Abbreviations:5-HIAA; 5-hydroxyindoleacetic acid, 3MI; 3-methylindole, 2IA; indole-3-aldehyde 3IA; indole-3-acetaldehyde, AA; anthranilic acid IA; indole-3-acetate, IAA; indole acrylic acid, KA; kynurenic acid, KYN; kynurenine, TRP; tryptamine, XA; xanthurenic acid.

Metabolites such as tryptamine (TRP), 3-methylindole (3MI), indole-3-acetaldehyde, IA, indole-3-aldehyde, indole acrylic acid, kynurenine, KA, XA and 5-hydroxyindoleacetic acid (5-HIAA) are ligands for AhR [56,57]. AhR is expressed throughout the body, but highest levels are found in the intestine, lung and skin, and it has been shown to regulate the immune system through several mechanisms [58] such as regulation of the differentiation of Th17 and Treg cells (see Figure 4) [59,60].

Indole and indole-3-acetamide are antagonist of PXR [61], which is most abundantly expressed in the liver. This intracellular receptor is involved in the down regulation of gluconeogenesis and lipid metabolism as well as immune regulation by decreasing inflammation via suppresion of the NF-kB pathway [62,63].

### 2.4. Serotonin and Melatonin Pathway 

**Figure 5:**
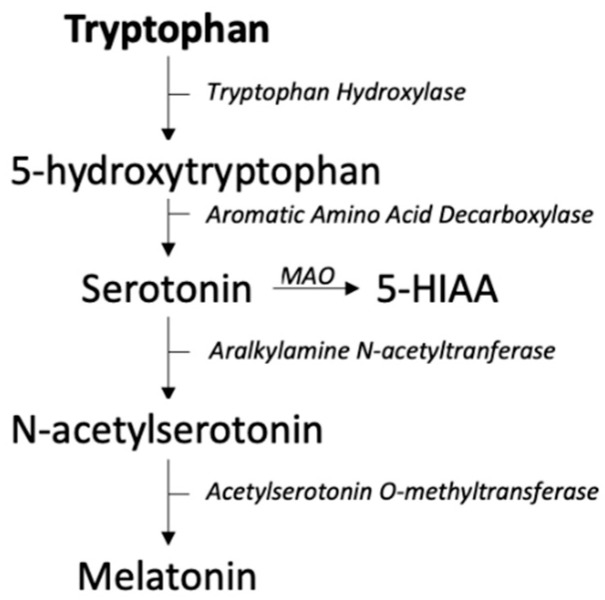
Serotonin pathway. Abbreviations: 5-HIAA; 5-hydroxyindoleacetic acid, MAO; monoamino oxidase.

The third pathway of tryptophan degradation leads to the formation of serotonin and melatonin, accounting for 1–2% of total degradation. The majority of serotonin (95%) is produced in the gastrointestinal tract by enterochromaffin cells where tryptophan is converted into 5-hydroxytryptophan by tryptophan hydroxylase 1 (TPH1), the rate limiting step, and consequently to serotonin by aromatic amino acid decarboxylase (see Figure 5) [64]. The microbiome and gut–brain axis seem to play an important role in the regulation of serotonin synthesis. Some gut bacterial species are known to produce serotonin in vitro, but whether this contributes to serotonin levels in mammals is not known [65]. In germ-free mice there is a lower expression of TPH1 and subsequently lower levels of serotonin in colon, feces and serum implicating a regulatory role of the gut microbiome in serotonin concentrations [66]. However, the underlying regulatory mechanism of the gut microbiome on serotonin remains unclear. From the gastrointestinal tract serotonin is transported via platelets to different peripheral sites such as the liver and cardiovascular system [67]. Serotonin synthesis also takes place in several cells such as immune cells (e.g., T-cells, B-cells, monocytes and macrophages) and pancreatic β-cells [61]. The effect of peripheral serotonin is dependent on the different receptors it is able to bind to [68].

The remaining production of serotonin occurs in the raphe nuclei located in the brainstem. Since tryptophan cannot cross the blood brain barrier (BBB), serotonin production in the brain is dependent on tryptophan uptake across the BBB, which is in part regulated by the concentrations of other large neutral amino acids (e.g., phenylalanine, valine) [69].

The final step in the serotonin pathway is the synthesis of melatonin in the pineal gland, but also in other sites throughout the body such as the liver, gastrointestinal tract and skin [70]. The regulation of melatonin is complex and beyond the scope of this review.

## 3. Tryptophan Metabolism in NAFLD 

### 3.1. Kynurenine Pathway

Inflammation has been regarded as one of the central mechanisms in NAFLD. Since the kynurenine pathway is upregulated by inflammatory molecules via IDO, it might play an essential role in the pathogenesis of NAFLD. In fecal samples of NAFLD patients, kynurenine levels were indeed increased while tryptophan levels were decreased, consistent with higher activity of the kynurenine pathway. Furthermore, microbiota were altered compared to healthy controls with an abundance of *Collinsella*, *Acinetobacter* and *Actinomyces* which were in turn related to higher levels of kynurenine [71].

To address the causality of this observation, the current literature is mainly dependent on pre-clinical studies. IDO-knock out mice on a high fat diet indeed displayed higher levels of macrophage markers and inflammatory molecules, such as IFN-γ, IL-1β and IL-6, compared to mice with IDO-activity. IDO knock-out mice also had an increased expression of fibrosis marker TGF-β2 and histology showed increased fibrosis in liver samples, suggesting that IDO-activity decreases inflammation and fibrosis [72]. Contrarily, in mice on a methionine and choline-deficient diet, which causes steatosis, with IDO-inhibition using by 1-methyl-D-triptophan, expression of pro-inflammatory genes coding for TNF-α and IL-1b were reduced as well as the expression of TGF-β and alpha smooth muscle actin (α-SMA), insinuating reduced fibrosis. These findings therefore suggest a that reduced IDO-activity would lessen inflammation and fibrosis [73].

When germ-free mice were transplanted with fecal samples from NAFLD patients there was an increase in kynurenine levels and in liver samples there was an increase of intrahepatic lipid accumulation [71]. These findings imply a crosstalk between the gut microbiome and the kynurenine pathway. Oral supplementation of kynurenine combined with a high fat diet in mice showed an increase in liver fat deposition and upregulation of CYP1A1, CYP450 and Scd1, associated with hepatic lipid metabolism [74].

In conclusion, increased kynurenine concentrations seem to stimulate lipid metabolism and intrahepatic fat deposition, whereas the role of IDO remains unclear.

### 3.2. Indole Pathway

Several inflammatory mechanisms in NAFLD are affected by metabolites of the microbe-derived indole pathway [75,76,77,78]. A potential driver of inflammation in NAFLD is the NF-kB pathway induced by the residential liver macrophages (Kupffer cells) [18,19]. In rodents, oral administration of indole after intraperitoneal injection of LPS reduced levels of cytokines Il-1β, Il-6 and Il-15, as well as levels of NF-kB [79]. In obese and lean mice oral suppletion of indole reduced the expression of Cd68, indicating a reduction in macrophage accumulation [80] which is consistent with earlier findings of indole reducing PFKFB3 in macrophages, which suppresses their proinflammatory state [81].

Another study showed that IA reduced macrophages in liver tissue and lowered levels of monocyte-chemoattractant protein 1 and TNF-a in mice with hepatosteatosis induced by a high fat diet [82]. In obese patients, lower IA levels were correlated with higher liver CT-values indicating NASH. After sleeve gastrostomy, IA levels increased in this population, while CT-liver values and fat-attenuation ameliorated, suggesting that IA has hepatoprotective properties [83].

Lastly, it was demonstrated that IPA can attenuate hepatic steatosis by increasing tight junction proteins and thus strengthening the gastrointestinal barrier, resulting in decreased circulating endotoxins and subsequently decreasing the activation of the NF-kB pathway via TLR4-activation [84]. Sehgal et al. found that lower circulating levels of IPA in obese patients without T2DM were inversely associated with fibrosis in liver biopsies. Furthermore, they showed that in vitro treatment of LX-2 cells with TGF-β1 and IPA reduced activity of stellate cells activated via the reduction of mRNA expression of COL1A2 and α-SMA, associated with hepatic stellate cell activation [85].

However, Liu et al. demonstrated that in chemokine ligand 4 (CCL4)-treated mice, oral administration of IPA resulted in an increased expression of α-SMA and COL1A2 suggesting in fact an opposite effect in vivo. Interestingly, they also found that CCL4-treatment reduced gut microbiome diversity which was reversed after treatment with IPA [86], indicating that IPA can modulate alterations in the gut microbiome induced by inflammation.

Since indole metabolites are produced by gut microbiota the above mentioned results underline the potential role of gut dysbiosis in the etiology of NAFLD with several studies indicating alterations of the gut microbiome in animals and humans with NAFLD [87].

### 3.3. Serotonin and Melatonin Pathways

In NAFLD patients increased levels of 5-HIAA, the serotonin marker commonly used to screen for neuroendocrine tumors, were correlated with an increased risk for liver related complications such as ascites and hepatocellular carcinoma [88].

In vitro, antagonism of serotonin and subsequently decreased levels of serotonin levels indeed resulted in lower expression of mRNA of fibrotic genes (α-SMA and COL1a1) and pro-inflammatory molecules (IL-1α and IL-8) [89]. In mice models with NAFLD, blocking serotonin receptors with tropisetron resulted in a reduction of steatosis and fibrosis in histological liver samples [90]. Another study found an increase in serotonin receptor 2A (HRT2A) in mouse livers with NAFLD and that in genetically deficient mice of the HRT2A receptor as well as antagonism of HRT2A reduced hepatic steatosis [83]. In this regard, melatonin has been studied in several contexts related to liver inflammation, for example by the induction of inflammation with ochratoxin [91]. In ducks, the oral suppletion of melatonin resulted in reduced serum levels of TNF-α, IL-1β, IL-6, but also in a decreased mRNA expression of TLR4 [92]. Similar results were found in mice [93], while injection of LPS in rats resulted in acute cellular infiltration in liver biopsies, which was alleviated by the oral administration of melatonin. This also resulted in lower lipid oxidation [94], which in turn is associated with the progression of NAFLD [95]. In patients with NAFLD treated with 5 mg of melatonin twice a day for 14 months there was significant reduction in cytokines IL-1, IL-6 and TNF-α compared to a placebo group, again suggesting the inflammation attenuating properties of melatonin [96].

Overall, serotonin seems to aggravate fibrosis and inflammation. This is also demonstrated by the increase of serotonin concentrations by selective serotonin reuptake inhibitors and a subsequently increase in NAFLD [97,98,99]. Even though melatonin is a downstream metabolite of serotonin, its effect seems to be contrary to the effect of serotonin, with melatonin exhibiting protective abilities against fibrosis and inflammation. The mechanisms which could determine a shift towards serotonin or melatonin are to date unknown.

## 4. Tryptophan and Metabolic Diseases

NAFLD is closely related to metabolic diseases such as diabetes, obesity, and atherosclerosis. Linking these diseases is a hyper-inflammatory state. Tryptophan and its metabolites attenuate the immune system in different ways [100,101,102,103,104] and are therefore involved in several pathological mechanisms in metabolic diseases.

### 4.1. Diabetes

In a large Finnish cohort of T2DM patients lower IPA-levels and higher CRP concentrations were found in patients with lower insulin secretion, suggesting a link between low-grade inflammation and T2DM, modulated by IPA [105]. Another study found that in patients with poor glycemic control, tryptophan concentrations were significantly lower compared to patients with moderate diabetes as well as healthy controls. They also found a higher kynurenine/tryptophan-ratio, both results indicating higher IDO-activity in patients with diabetes, which is upregulated by inflammatory molecules [106]. Another study confirmed the results and even found that a higher kynurenine/tryptophan-ratio was associated with higher mortality in T2DM [107].

In rats it was demonstrated that multiple enzymes involved in the kynurenine pathway are expressed in pancreatic β-cells, but the physiological role has not been discovered [108]. In mice, it was discovered that tryptophan (and phenylalanine) selectively bind to GPR142, a G-coupled receptor with a high expression in pancreatic cells and the gut, and could therefore modulate the secretion of insulin and glucagon-like peptide 1(GLP-1) [109].

Although the link between inflammation, an upregulation of IDO-activity and T2DM seems to be established and is in a similar direction as NALFD, the underlying molecular pathways in which different metabolites such as indoles are involved in glycemic control, and thus the relation with the gut microbiome as well, remains the be elucidated.

### 4.2. Obesity

A similar relation between low-grade inflammation and (over-)activity of the kynurenine pathway as in NALFD is also found in patients with obesity. As in patients with T2DM, tryptophan concentrations were found to be lower in patients with obesity compared to healthy controls as well as having a higher kynurenine/tryptophan-ratio. On the other hand, serum concentrations of IAA, IPA, IAAa and indoxyl sulfate were lower in obese patients and increased concentrations of CRP and IL-6 were associated with lower levels of indole metabolites [110]. This again indicates that IDO-activity is induced by a pro-inflammatory state increasing the activity of the kynurenine pathway, whereas the formation of indole metabolites is decreased.

One study found that blocking AhR reduced obesity in mice on a Western diet. They proposed that low-grade inflammation increases IDO-activity, thus directly increasing kynurenine concentrations. Since kynurenine is an AhR-agonist, overstimulation of AhR by higher concentrations of kynurenine might lead to obesity [111]. Interestingly, in obese IDO knock out mice there is an apparent shift towards the indole pathway compared to wild type mice with increased IDO-activity in the gut and interestingly higher levels of IA [112], possibly indicating an IDO-dependent formation of indoles. 

### 4.3. Atherosclerosis

The role of the kynurenine pathway in atherosclerotic disease has been extensively reviewed [113,114,115]. A state of low-grade inflammation in atherosclerosis is marked by increased levels of IL-6, an inducer of IDO [116]. Once more, research shows that decreased tryptophan and an increased kynurenine/tryptophan-ratio is associated with the severity of atherosclerosis [113] and thus indicating that increased IDO-activity is related to this state of low-grade inflammation.

One observational study, in a cohort with patients undergoing carotid endarterectomy, bypass surgery in a limb or amputation due to ischemia, found indeed that the plasma kynurenine/tryptophan-ratio was related to advanced atherosclerosis as well as to a higher risk of post-operative cardiac complications. However, they also demonstrated that serum concentrations of IPA and indole-3-aldehyde were lower in patients with severe atherosclerosis [117] which is in agreement with research implicating a protective role against inflammation by the indole pathway.

Another study investigated the effect of antibiotics on the development of atherosclerosis in mice. Administration of antibiotics resulted in a decrease of Bacteroidetes and Clostridia species, both of which are associated with tryptophan metabolism in the gut and they demonstrated that a reduction in these species was related to a decrease in gut-derived tryptophan metabolites [51,118]. Moreover, they demonstrated that tryptophan suppletion after antibiotics could reduce aortic lesion size in these mice, although the relation with atherosclerosis was not significant [118]. This demonstrates that perturbations in the gut microbiome alter tryptophan metabolite production, and this has a measurable effect in vascular disease.

It is important to note that due to the wide spectrum of metabolic diseases there are several other conditions that share etiological factors with NAFLD and are also associated with metabolic diseases. The interaction between tryptophan metabolism and such diseases remains to be further elucidated. For example, in the case of obstructive sleep apnea (OSA) there are some interesting papers regarding the relationship between tryptophan metabolism and OSA [119,120]. However, further research is needed to establish potential correlations and underlying mechanisms.

## 5. Conclusions and Future Perspectives

Tryptophan metabolism constitutes a complex network of human and gut microbial metabolites interacting with several physiological and pathological processes. The extent and implications of these interactions are not completely elucidated, but as described in this review, they seem of vital importance for immunological response, fibrosis, glycemic control, lipid metabolism and hormonal homeostasis. In the spectrum of cardiometabolic diseases, there seems to be an imbalance in the regulation of the different (intestinal vs. endogenous) pathways following the degradation of tryptophan. This may be due to an increased activity of the kynurenine pathway, indicated by higher IDO-activity and increased concentrations of associated downstream metabolites, which is linked to increased inflammation and fibrosis and therefore linked to metabolic diseases such as NAFLD.

Furthermore, it has been demonstrated that IDO-activity is a key regulator in this process and that there is an active shift increasing the kynurenine metabolites and decreasing indole metabolites. The indole pathway, however, is indicated to have anti-inflammatory properties, particularly in NAFLD and atherosclerosis. Therefore, increasing metabolite concentrations this pathway is of therapeutic interest.

This imbalance is also prominent in the serotonin pathway, where serotonin seems to aggravate NAFLD and suppletion of melatonin decreases inflammation and fibrosis.

We therefore propose that in NALFD the balance between the IDO, indole and serotonin pathways has been tipped towards the pro-inflammatory downstream actions of tryptophan metabolism. Restoring this balance (e.g., via microbiota manipulation) may be a major step forward to prevent the progression of NAFLD towards NASH and to weaken the associations between NALFD, T2DM and cardiovascular disease in obesity. To this end, more fundamental research is needed to develop therapeutic strategies decreasing the effect of tryptophan on a low-inflammatory state in metabolic diseases and thereby possibly reducing cardiovascular and metabolic complications.

## Glossary

3-MI	3-methyl indole
5-HIAA	5-Hydroxyindoleacetic acid
α-SMA	Alpha Smooth Muscle Actin
AA	Anthranilic Acid
AA0	Neutral Amino Acid
ACE2	Angiotensin converting enzyme 2
AhR	Aryl hydrocarbon receptor
BBB	Blood brain barrier
CCL-4	Chemokine Ligand 4
GC	Glucocorticosteroids
GLP1	Glucagon-Like Peptide 1
IA	Indole-3-acetate
IDO	Indoleamine 2,3-dioxygenase
IL	Interleukin
INF-γ	Interferon γ
IPA	Indole-3-propionate
KA	Kynurenic acid
KYN	Kynurenine
LPS	Lipopolysaccharide
MAFLD	Metabolite Associated Fatty Liver Disease
MEL	Melatonin
NAD	Nicotinamide Adenine Dinucleotide
NAFLD	Non-Alcoholic Fatty Liver Disease
NASH	Non-Alcoholic SteatoHepatitis
PA	Picolinic Acid
PXR	Pegnane X receptor
TDO	Tryptophan-2,3-dioxygenase
TLR4	Toll Like Receptor 4
TNF	Tumor Necrosis Factor
TPH1	Tryptophan Hydroxylase 1
Trp	Tryptophan
T2DM	Type 2 Diabetes
XA	Xanthurenic acid
